# Systematic Review of Programs Treating High-Need and High-Cost People With Multiple Chronic Diseases or Disabilities in the United States, 2008–2014

**DOI:** 10.5888/pcd12.150275

**Published:** 2015-11-12

**Authors:** Sara N. Bleich, Cheryl Sherrod, Anne Chiang, Cynthia Boyd, Jennifer Wolff, Eva DuGoff, Claudia Salzberg, Keely Anderson, Bruce Leff, Gerard Anderson

**Affiliations:** Author Affiliations: Sara N. Bleich, Cheryl Sherrod, Anne Chiang, Jennifer Wolff, Eva DuGoff, Claudia Salzberg, Keely Anderson, Gerard Anderson, The Johns Hopkins Bloomberg School of Public Health, Baltimore, Maryland; Cynthia Boyd MD, Bruce Leff, The Johns Hopkins University School of Medicine, Baltimore, Maryland.

## Abstract

**Introduction:**

Finding ways to provide better and less expensive health care for people with multiple chronic conditions or disability is a pressing concern. The purpose of this systematic review was to evaluate different approaches for caring for this high-need and high-cost population.

**Methods:**

We searched Medline for articles published from May 31, 2008, through June 10, 2014, for relevant studies. Articles were considered eligible for this review if they met the following criteria: included people with multiple chronic conditions (behavioral or mental health) or disabilities (2 or more); addressed 1 or more of clinical outcomes, health care use and spending, or patient satisfaction; and compared results from an intervention group with a comparison group or baseline measurements. We extracted information on program characteristics, participant characteristics, and significant (positive and negative) clinical findings, patient satisfaction, and health care use outcomes. For each outcome, the number of significant and positive results was tabulated.

**Results:**

Twenty-seven studies were included across 5 models of care. Of the 3 studies reporting patient satisfaction outcomes, 2 reported significant improvements; both were randomized controlled trials (RCTs). Of the 14 studies reporting clinical outcomes, 12 reported improvements (8 were RCTs). Of the 13 studies reporting health care use and spending outcomes, 12 reported significant improvements (2 were RCTs). Two models of care — care and case management and disease management — reported improvements in all 3 outcomes. For care and case management models, most improvements were related to health care use. For the disease management models, most improvements were related to clinical outcomes.

**Conclusions:**

Care and case management as well as disease management may be promising models of care for people with multiple chronic conditions or disabilities. More research and consistent methods are needed to understand the most appropriate care for these high-need and high-cost patients.

## Introduction

The number of high-need and high-risk Americans with multiple chronic conditions or disabilities is large and is increasing; more than one-quarter of adults have at least 2 chronic conditions (1). Caring for all these people is expensive and accounts for 84% of total US health care spending (2,3). People with multiple chronic conditions are at greater risk for disability, activity limitations (such as difficulty walking) (2), mortality, poor functional status, unnecessary hospitalizations, adverse drug events, among many other challenges (2,4–7) than those with 1 or no chronic conditions. They also tend to be higher users of medical care services than those with 1 or no chronic conditions (2). The problem of multiple chronic conditions is not restricted to adults. More than 6 percent of children have more than 1 chronic condition and experience higher rates of activity limitations (such as school absences due to sickness) than children with 1 chronic condition (2).

Finding ways to provide better and less expensive care for people with multiple chronic conditions is a pressing public health and medical concern. Evaluations of programs treating this population are beginning to emerge in peer-reviewed literature. One systematic review conducted in 2009 identified 15 successful models of care (ie, care and case management) for adults with chronic disease that improved at least 1 health outcome (eg, quality of life, quality of care, cost of health services), although it did not specifically focus on programs targeting patients with multiple chronic conditions (8). This review (8) examined studies conducted in 2008 and earlier. 

Other reviews focused on specific interventions, such as the core characteristics for the geriatric emergency management model (9); the effect of home-based primary care for homebound senior adults on individual, caregiver, and systems outcomes (10); the effectiveness of interventions designed to improve outcomes for patients with multiple chronic conditions in primary care and community settings (11), and characteristics of comprehensive care programs for patients with multiple chronic conditions (12). A better understanding of programs that focus on patients with multiple chronic conditions is needed to inform clinical guidelines (13). The aim of our study was to perform an updated systematic review of care models for high need and high risk people with multiple chronic conditions or disabilities.

Our review is timely because health care is shifting from a volume-based to a value-based ethos under the Affordable Care Act (ACA) (14), and because the ACA established a new office to coordinate care for people who are eligible for both Medicaid and Medicare (“dual eligibles”), a population with high rates of multiple chronic conditions (15). Our review contributes to the literature by focusing on how recent interventions directed to people with multiple chronic conditions affected clinical outcomes, patient satisfaction, and health care use or costs.

## Methods

We used the methods recommended by the Agency for Healthcare Research and Quality Methods Guide for Effectiveness and Comparative Effectiveness Reviews (16).

### Data source

We searched Medline for relevant studies published from May 31, 2008, through June 10, 2014. The start date was selected based on the final date used in the prior systematic review that focused on successful models of care for adults with chronic disease (8). We developed a Medline search strategy based on medical subject heading terms and text words of key articles that we identified a priori.

The search strategy relied on medical subject headings (MeSH) and text word field descriptions. The search combined the MeSH term “outcome and process assessment (health care)” with terms describing types of interventions (eg, patient-centered medical home, transitional care, geriatric evaluation and management, program of all-inclusive care for the elderly, behavioral medicine, self-management, pharmaceutical services, palliative care, disease management, case management, substance abuse, and behavioral medicine) and the health care setting (eg, hospitals, nursing homes, emergency care, rehabilitation centers, and home care services). We used a snowball sampling approach by reviewing the reference lists for all included articles and relevant review articles to identify additional articles that the database searches may have missed.

### Study selection

Articles were considered eligible for this systematic review if they met the following criteria: a) included people with multiple chronic conditions (behavioral or mental health) or 2 or more disabilities; b) were designed to address 1 or more of the following domains: improvement in clinical outcomes, efficiency of health care use and spending, or patient satisfaction; c) compared results from an intervention group with a comparison group or with baseline measurements; d) described results conducted in randomized controlled trials (RCTs), quasi-experimental studies, or natural experiments; e) were conducted in the United States (to maximize generalizability to the US health care context); and e) were published in English. We excluded studies whose model focused on a single chronic condition or a single disability.

Two authors (C.E.S., K.A.) read each title and abstract to assess how well they fulfilled the inclusion criteria for full text review. Authors also hand-searched bibliographies of relevant articles to identify additional articles.

### Data extraction

Data for the included studies were extracted independently by 2 authors (K.A., C.E.S.) and checked for consistency with the published article. Before data entry, the data abstraction approach was pilot tested with 5 articles. The abstraction forms were used to collect data related to program characteristics, participant characteristics, and significant clinical findings, patient satisfaction, and health care outcomes. Both significant and nonsignificant outcomes were collected if they related to 1 of 3 outcomes: clinical (eg, mortality, functional status, pain), patient satisfaction, or health care use or spending (eg, hospitalization, nursing home admission, net savings). Our definition of clinical outcomes included measures reported by the patient and measures reported about the patient (eg, depression, hemoglobin A1c) and did not include measures of patient satisfaction.

Included studies were categorized by program type and outcomes (clinical, patient satisfaction, and health care use or costs). The program type and outcomes were qualitatively coded by 2 authors and adjudicated by a third, where necessary. For each outcome, we tabulated the number of significant results in the total number of outcomes reported for that domain. Because of the limited number of studies for each type of intervention, and because the populations differed considerably on multiple dimensions, we did not quantitatively pool the results.

## Results

### Literature search

Our search identified 1,736 potential articles (Figure). Of these, 145 articles met all criteria on the basis of title and abstract screening. Of these, 89 had an ineligible target population, 22 lacked an intervention, and 7 reported no outcomes. A total of 27 articles met all our criteria.

**Figure:**
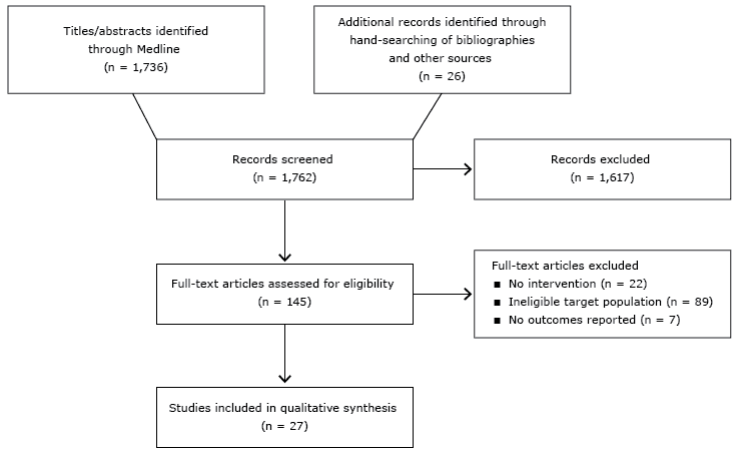
Flow diagram of article selection.

### Study characteristics and intervention

The interventions included in this review focused on improving health outcomes among high-need and high-cost people with multiple chronic conditions or disabilities by using strategies to manage the chronic health condition(s). The practice settings of the 27 studies varied, from the home to medical centers or hospitals to nursing facilities (Table 1). Of the 27 studies, 12 were RCTs, and the methodology of the remainder was either quasi-experimental designs or natural experiments. All interventions involved multiple components, such as coupling medication management with nutritional consultations. Each intervention enrolled different target populations.

**Table 1:** Characteristics of Studies Testing the Effect of Programs Treating High-Cost, High-Needs People (N = 27) by Study Type, United States, May 31, 2008–June 10, 2014

First Author, Year, State(s)	Practice Setting	RCT	Study Design for non-RCTs	Sample Size	Target Population	Program Type: Intervention Description
Alexopoulos (32), 2011, California, New York	Academic medical center	Yes	—	221	Adults >59 years with major depression and executive dysfunction	CDSM: Problem-solving therapy in 12 weekly sessions in which participants set goals, proposed ways to reach them, created action plans, and evaluated the accomplishment of their goals.
Barrett (17), 2010, Ohio	Hospital	No	Longitudinal; participants compared with themselves over time (no control group)	585	High-risk older adults (≥60 years) in the community	CM: Proactive gatekeeper program and case management model used to identify at-risk older adults in the community; nonclinician volunteers underwent 1-hour to 2-hour training to recognize signs and symptoms indicating that patient needed assistance to remain safe and independent in the community.
Blank (18), 2011, Pennsylvania	Academic medical center	Yes	—	238	Patients with HIV and serious mental illness	CM: Care assigned to an advanced-practice nurse who provided in-home consultations and coordinated medical and mental health services for 1 year according to a disease management model. The nurse collaborated with prescribing providers, pharmacists, and case managers to organize medication regimens and coping mechanisms for barriers to medication adherence.
Boult (19), 2011, Maryland	Community-based primary care practices	Yes	—	850	Patients aged ≥65 years at high risk of using health services	CM: Guided care: a comprehensive assessment, evidence-based care planning, monthly monitoring of symptoms and adherence, transitional care, coordination of health care professionals, support for self-management, support for family caregivers, and enhanced access to community services.
Casey (20), 2011, Arkansas	Tertiary care children's hospital	No	Pre/post (no control group)	255	Medically complex children (<18 years) with at least 2 chronic medical conditions	CM: Improved coordination of care with PCPs, subspecialists, hospitalists, and community-based services.
Comart (42) , 2013, Massachusetts	Long-term care	No	Case-control	250	Frail, medically complex seniors (≥65 years)	NH: An interdisciplinary consult team formed to facilitate conversations about goals of palliative care; the team consisted of a PCP, clinical nurse specialist, chaplain, social worker, and a psychologist, who also served as the lead administrator for the program.
De Jonge (21)2014, Washington, DC	Home-based primary care	No	Case-control	2,883	Frail and elderly (≥65 years) Medicare beneficiaries	CM: A mobile care team that delivered medical services to homebound elders with disabling and multiple chronic conditions. The interprofessional team consisted of physicians, nurse practitioners, geriatricians, social workers, and other health care providers to provide case management and other services.
Edelman (35), 2010, Virginia, North Carolina	Veterans Affairs medical center	Yes	—	239	Adults of any age with poorly controlled diabetes and hypertension	DM: Group medical clinics that comprised 7 to 8 patients and a care team consisting of a primary care general internist, a pharmacist, and a nurse or other certified diabetes educator. Each session included structured group interactions moderated by the educator; the pharmacist and physician adjusted medication to manage each patient’s hemoglobin A1c level and blood pressure.
Edes (22), 2014, United States	Home- based primary care	No	Difference in difference	9,425; 31 interviews conducted for qualitative analysis	Veteran Medicare beneficiaries with multiple chronic conditions	CM: Interdisciplinary teams of physicians, nurses, social workers, dietitians, pharmacists, and other health care providers working together to deliver comprehensive care services. Care services used a single-care plan with medication reconciliation and caregiver training and other practices. The program focused on those beneficiaries with multiple complex chronic conditions for whom routine clinic-based care has not been successful and effective.
Friedman (23), 2009, New York, Ohio, West Virginia	Home visits	Yes	—	766	High-risk Medicare beneficiaries with disability and recent significant health care use	CM: A primary care-affiliated disease management and health promotion nurse intervention among Medicare beneficiaries with disabilities; consisted of monthly home visits by trained nursing staff who coordinated with the primary care provider, made referrals to community resources, and set goals with patients and caregivers for the following areas: telephone use, shopping, ordinary housework, money management, medication management, and meal preparation.
Gellis (36), 2012, New York	Home health care	Yes	—	115	Homebound older adults with heart failure or chronic respiratory failure	DM: A telehealth monitoring system that allowed patients to report vital signs daily and enhance self-management of their medical conditions through counseling and education.
Gutgsell (37), 2013, Ohio	Hospice	Yes	—	200	Adult palliative care patients	DM: Palliative care incorporating 20-minute music therapy intervals administered according to prespecified pain control protocol.
Jerant (33), 2009, California	Academic Medical Center	Yes	—	415	Adults (≥40 years) with 1 or more of 5 common chronic illnesses and functional impairment	CDSM: Home-based, peer-led, self-management training where individuals participated in 6 weekly sessions (via a home visit or telephone call) lasting approximately 60 minutes to 70 minutes led by a nonclinician peer using a standardized curriculum. The aim of the groups was to teach fundamental self-management tasks.
Kiosses (34), 2011, New York	Home	No	Case study (no control group)	2	Depressed, cognitively impaired, disabled elderly (≥65 years	CDSM: Problem adoption therapy (PATH) delivered by 12 in-home sessions conducted weekly, initial assessment, and a personalized treatment plan.
Kuo (24), 2013, Arkansas	Academic medical center	No	Pre/post (no control group)	120	Medically complex children (<18 years)	CM: Improved coordination of care with PCPs, subspecialists, hospitalists, and community-based services.
Li (25), 2013, New York, Ohio, West Virginia	Home visits	Yes	—	499	Medicare recipients needing or receiving help with at least 3 IADLs or 2 ADLs, who had recent significant health-care use	CM: Monthly home visits by trained nursing staff who coordinated with PCP, made referrals to community resources, and set goals with patients and caregivers for the following areas: telephone use, shopping, ordinary housework, money management, medication management, and meal preparation.
Luptak (26), 2010, Utah	Home telehealth	No	Pre/post (no control group)	132	Rural veterans aged ≥65 years with high use of health care services	CM: A Care Coordination Home Telehealth intervention consisting of face-to-face orientation, telephone contact with a designated care coordinator, and daily monitoring sessions using an in-home telehealth device to assess participants’ medication usage, compliance, and symptoms and to provide patient education.
Moggi (38), 2010, California	Substance use disorder programs affiliated with the Veterans Affairs	No	Pre/post (no control group)	132	Adults of all ages with substance abuse and personality disorders	DM: A representative sample of 15 substance use disorder programs affiliated with the US Department of Veterans Affairs selected on the basis of criteria such as large patient pool, geographic dispersion, and representative treatment orientations.
North (27), 2008, Colorado	Veterans Affairs Medical Center	No	Pre/post (no control group)	104	Frail, chronically ill, homebound, elderly (≥65 years) veterans	CM: Home visits, coordinated care, and referral to community resources.
Ornstein (28), 2013, New York	Home health care	No	Longitudinal with assessments at 3 weeks and 12 weeks (no control group)	140	Homebound adults of all ages receiving palliative care	CM: A comprehensive initial home visit and assessment by a physician with subsequent follow-up care, interdisciplinary care management including social work, and urgent in-home care as necessary.
Ouslander (29), 2009, Georgia	Nursing home	No	Pre/post (no control group)	289 (beds)	Nursing homes with the highest hospitalization rates	CM: Prospective quality improvement initiative conducted by the Georgia Medical Care Foundation, the Medicare Quality Improvement Organization for Georgia. Participating NHs were provided with communication and clinical practice tools and strategies designed to assist in reducing potentially avoidable hospitalizations, and on-site and telephonic support by an advanced practice nurse.
O'Toole (30), 2009, Rhode Island	Veterans Affairs Medical Center	No	Retrospective cohort study with assessments at 6 months at 12 months (control group)	177	Homeless adult veterans of all ages	CM: Chronic care model used to assign a PCP and a nurse case-manager; on-site integration of homeless-specific services, fixed day schedule for drop-in care and follow-up, patient assessment, outreach and coordination of care with community shelters, standard patient educational material, and access to self-management classes.
Petry (39), 2010, Connecticut	HIV drop-in center	Yes	—	170	HIV-positive adults of all ages with cocaine or opioid use disorders	DM: A group-based contingency management intervention that rearranged the environment to frequently detect behaviors targeted for change using group sessions, weekly breath samples (screened for alcohol), and urine specimens (screened for opioids); opportunities for prizes for completing group and having substance-free specimens.
Sorocco (31), 2013, Oklahoma	Veterans Affairs Medical Center	No	Longitudinal with assessments at 3 months and 6 months (no control group)	6	Elderly (≥65 years) veterans with complex medical conditions and their caregivers	CM: A home telehealth monitoring system where patients provided daily vital signs and were supervised by an interdisciplinary treatment team.
Takahashi (43), 2013, Minnesota	Academic medical center	No	Prospective cohort study (control group)	40	Medically complex adult (>60 years) patients with a high risk of readmission based on Elder Risk Assessment	TC: A care transition team (nurse practitioner, case manager registered nurse, PCP, and consulting geriatrician) providing care coordination and an in-home visit 1 to 3 days after discharge
Wakefield (40), 2011, Iowa	Veterans Affairs Medical Center	Yes	—	302	Veterans of all ages with diabetes and hypertension	DM: Close surveillance via a home telehealth device (to monitor blood glucose and blood pressure) and nurse care management over a 6-month time period. A high-intensity group received tailored health information tips and questions; a low-intensity group responded to 2 daily questions but did not receive information tips and questions given to the high-intensity group. The primary goal of the study was clinical outcomes of hemoglobin A1c and systolic blood pressure.
Wakefield (41), 2012, Iowa	Veterans Affairs Medical Center	Yes	—	302	Veterans of all ages with diabetes and hypertension	DM: Close surveillance via a home telehealth device (to monitor blood glucose and blood pressure) and nurse care management over a 6-month time period. A high-intensity group received tailored health information tips and questions; a low-intensity group responded to 2 daily questions but did not receive the information tips and questions given to high-intensity group. The study reported on secondary outcomes, such as medication adherence and self-efficacy scores.

Abbreviations: ADL, activities of daily living; CM, care or case management; CDSM, chronic disease self-management; DM, disease management; IADL, instrumental activities of daily living; NH, nursing home; PCP, primary care provider; TC, transitional care; —, not applicable.

### Summary of evidence by model of care

In our summary of intervention model types (eg, case management, care transitions) and outcomes (Table 2) the numerator is the number of studies showing a significant improvement (*P* < .05 for reported outcomes), and the denominator is the number of studies in which this outcome was assessed. The specific outcomes for each study are reported in the Appendix, Table 1 (patient satisfaction outcomes), Table 2 (clinical outcomes), and Table 3 (health care utilization and spending). The 27 studies reported on 5 model types, 4 of which yielded successful outcomes: care or case management, chronic disease self-management, disease management and nursing home (no significant outcomes were reported for the transitional care model).

**Table 2:** Summary of Evidence From 27 Successful Studies Testing the Effect of Programs Treating High-Cost, High-Needs People (N = 27) by Model Type, United States, May 31, 2008–June 10, 2014

Model	Outcome			
	Study Type and Number	Patient Satisfaction^a^	Clinical^a^	Health Care Use^a^
Care and case management	3 RCTs, 9 quasi-experimental, 1 case-control, 1 prospective cohort	1/2	4/4	8/9
Chronic disease self-management	2 RCTs, 1 case study	—	1/3	—
Disease management	6 RCTs, 1 quasi-experimental	1/1	5/6	1/1
Nursing home	1 Case-control	—	1/1	1/1
Transitional care	1 Quasi-experimental	—	—	0/1

Abbreviations: RCT, randomized controlled trial; —, not applicable.

a The numerator is the number of studies showing a difference in outcome, and the denominator is the number of studies in which this outcome was assessed.

#### Care or case management

Care or case management is a collaborative model in which a nurse or social worker helps patients with multiple chronic conditions and their families to assess problems, communicate with health care providers, and navigate the health care system (8). We identified 15 studies in which care or case management was used to care for patients with multiple chronic conditions (17–31). We report only studies with significant positive outcomes. The other studies had insignificant results, negative results, or did not report any of the 3 study outcomes (patient satisfaction, clinical outcomes, health care use and spending). Of the 15 studies, 1 (23) reported successful patient satisfaction outcomes, 4 reported successful clinical outcomes (18,24,25,28), and 8 reported successful health care use and spending outcomes (8,17,20–22,24,27,29,30).

**Successful patient satisfaction outcomes.** Friedman et al (23) evaluated the impact of a primary care nurse intervention for disease management and health promotion on patient satisfaction among disabled Medicare beneficiaries’ (RCT).

**Successful clinical outcomes.** Blank et al (18) assessed the impact of in-home consultations provided by an advanced practice nurse on the viral load among patients with HIV and serious mental illness (RCT). Kuo et al (24) evaluated parents’ perception of health related quality of life after enrolling their children with complex medical conditions in a 12-month Medical Home Clinic for Special Needs Children program (non-RCT). Li et al (25) assessed the effects of home visiting nurse interventions on activities of daily living (RCT). Ornstein et al (28) assessed the effects of a home-based primary and palliative care program on homebound patients (non-RCT). 

**Successful health care use and spending outcomes.** Barrett et al (17) assessed the effects of the Gatekeeper program, a program designed to provide case management and a link to community-based resources to at-risk elderly adults (non-RCT). Boult et al (8) studied the effects of guided care teams on the use of health service by adults aged 65 and older with multiple chronic conditions (RCT). Casey et al (20) evaluated the effects of using multidisciplinary teams to provide coordinated care to children with medically complex conditions (non-RCT). De Jonge et al (21) assessed the effects of home-based primary care on frail and elderly Medicare beneficiaries (non-RCT). Edes et al (22) examined the impact of a home-based primary care program delivering comprehensive primary care to patients at home (non-RCT). The Kuo et al (24) intervention described above also evaluated parents’ perception of health care delivery and outcomes after enrolling their children with complex medical conditions in a 12-month Medical Home Clinic for Special Needs Children program (non-RCT). North et al (27) assessed the impact of a home-based primary care program managed by nurse-practitioners that provided care coordination (non-RCT). O’Toole et al (30) evaluated the impact of patient-centered medical homes on health care access for 4 high-risk groups: homeless veterans, cognitively-impaired elderly, women veterans, and patients with serious mental illness (non-RCT).

#### Chronic disease self-management

Chronic disease self-management programs are structured, time-limited interventions designed to provide health information to patients and engage them in actively managing their chronic conditions (8). We identified 3 studies that used chronic disease self-management to care for patients with multiple chronic conditions (32–34). Of these, only 1 study (32) reported successful clinical outcomes.

**Successful clinical outcomes.** Alexopoulos et al (32) assessed whether problem-solving therapy reduces disability more than supportive therapy in older patients (≥59 y) with depression and executive dysfunction (RCT).

#### Disease management

Disease management programs supplement primary care by providing patients with information about their chronic conditions in writing or by telephone (8). We identified 7 studies that used disease management to care for patients with multiple chronic conditions (35–41). Of these, 1 study reported successful patient satisfaction outcomes (36), 5 studies reported successful clinical outcomes (36–40), and 1 study reported successful health care use and cost outcomes (35).

**Successful patient satisfaction outcomes.** Gellis et al (36) conducted an RCT evaluating the outcomes and effectiveness of a telehealth intervention among homebound elderly adults with complex chronic conditions.

**Successful clinical outcomes.** The Gellis et al (36) intervention described above also assessed depression as an outcome (using the Center for Epidemiologic Studies Depression scale and the Patient Health Questionnaire), which had significant improvements. Gutgsell et al (37) conducted an RCT to determine the efficacy of music therapy to reduce pain for people receiving palliative care. Petry et al (39) evaluated the effect of contingency management treatments among HIV-positive patients with cocaine or opioid use disorders who attended a drop-in center for care (RCT). Wakefield et al (2011) (40) used an RCT to test whether a nurse-managed home telehealth intervention improved health outcomes among veterans with diabetes mellitus and hypertension.

**Successful health care use and cost outcomes.** Edelman et al (35) used an RCT to test the effectiveness of group medical clinics in the management of diabetes mellitus and hypertension. The Moggi et al (38) study described above also examined the outcomes of remission and hospitalization (non-RCT).

#### Nursing home

Several models were developed to improve the care of nursing home residents (8). Most rely on primary care provided by an advanced-practice nurse or physician assistant. In one study, we observed significant improvements in 2 domains (clinical, health care use and spending): Comart et al (42) assessed whether a palliative care consult service in long-term care settings provided participants with more favorable treatments and better clinical outcomes than a control group (ie, residents who received care before the palliative care consult service was implemented) (non-RCT).

#### Transitional care

Most interventions in transitional care were designed to facilitate smoother, safer, and more efficient transitions from the hospital to the next site of care: another healthcare setting or home (43). A nurse or an advanced-practice nurse who prepares the hospitalized patient and informal caregiver for the transition usually leads transitional care interventions. We observed no significant health care use or cost outcomes for this model.

## Discussion

This systematic review identified 27 studies published from 2008 through 2014 that met our inclusion criteria across 5 models of care. Of the 27 studies focusing on high-need and high-cost people with multiple chronic conditions or disabilities, many did not show significant improvement on any of the triple aims (ie, reducing spending, improving clinical outcomes, or increasing satisfaction). This is especially surprising given the expected publication bias of reporting only favorable results.

Of the 27 studies, 2 (23,36) of the 3 (23,26,36) reporting patient satisfaction outcomes observed significant improvements (both were RCTs); 12 (18, 25,28,32–33, 34,36–40, 42) of the 14 (18, 25,28,32–34,36–42) reporting clinical outcomes observed significant improvements (8 [18,25,32-33, 36-37, 39-40] were RCTs); and 12 (17,19–22,24,27,30,35,38,42,43) of the 13 (17,19–22,24,27,29,30,35,38,42,43) reporting health care use and spending outcomes observed significant improvements (2 [19, 35] were RCTs). The strength of the evidence is relatively stronger for the outcomes of patient satisfaction and clinical outcomes where the dominant study design is RCT compared with non-RCT. In contrast, the evidence base for the health care use and spending outcomes was less robust, because it relied primarily on nonrandomized evaluations with less rigorous study designs. We cannot discern if this is the result of study design or some other factor.

Achieving success on multiple dimensions was even more elusive, although few studies reported on each of the triple aims. No study reported significant improvements in all 3 study outcomes, and only 2 studies reported significant improvements in 2 study outcomes (36,42). This could be because most studies reported outcomes for only 1 dimension. The reported outcomes varied substantially across studies and domains making comparisons difficult. It would be helpful if these types of initiatives used a standard set of indicators to facilitate assessment of common factors that promote success.

Only 2 models — care and case management and disease management — reported improvements in all 3 outcomes. However, these outcomes were not in the same study but across multiple studies. Of note, within each model, the actual processes or applications varied widely since heterogeneity, complexity, and multicomponent are hallmarks of these types of programs (44). For care and case management models, most improvements were related to health care use (8,17,20–22,24,27,29,30). For the disease management models, most improvements were related to clinical outcomes (36–41). This makes it challenging to discern what attributes are common to successful programs.

People with multiple chronic conditions account for 84% of all health spending in the United States (2,3); however, relatively few studies published in peer reviewed literature assessed performance of programs designed explicitly to address the needs of this population despite the multitude of these programs. Robust evidence on the care of patients with multiple chronic conditions is limited, and interventions to date had mixed effects. The limited evidence on the effectiveness of these programs suggests that they are likely to be more effective if targeted at risk factors or specific functional difficulties (11,45).

The studies included in this review used different methodologies (eg, RCT, quasi-experimental study). They studied different heterogeneous patient populations and different models of care developed by a variety of entities (eg, government, insurers, providers). Going forward, large-scale changes in health care delivery resulting from the Affordable Care Act underscore the growing importance of understanding both the characteristics of the model of care and the context of the environment in which it is implemented. The limited available evidence from the peer reviewed literature included in this study and prior reviews (46) points to care and case management models as a more effective approach for reducing health care use and costs among high-cost, high-need patients with multiple chronic conditions.

We observed that a substantial proportion of the successful programs relied on the home as the location for the intervention (17,21–23,25,27,28,33,34,36,40,41). People who are home-limited because of multiple chronic conditions and functional impairment are challenged in accessing usual primary care services and are among the most costly patients to the health care system. Clinical interventions such as home-based primary care may be effective in meeting the triple aim via several mechanisms. These programs effectively target a costly and vulnerable population. Furthermore, these programs use interdisciplinary care teams to address both the medical and social needs of their patients (47).

The findings of this study are similar to the 2009 systematic review (8) suggesting that little has changed in the last 6 years. There are also systematic reviews of specific programs that reach similar conclusions. For example, a recent systematic review by Conroy et al (48) of 5 RCTs (all 5 of which are included in our study) assessed the impact of comprehensive geriatric assessment models on frail older people who were admitted to acute hospital settings and who are discharged home within a short period. The study found no clear evidence of the benefit for these interventions in terms of mortality, readmissions, or subsequent institutionalization, functional ability, quality of life, or cognition.

More research is needed in several areas including the comparative effectiveness of different models of care, overall and for common chronic disease clusters (49), as well as to determine whether the impact of programs differs by patient characteristics (eg, number of chronic conditions, presence of a comorbid disability, presence or absence of a mental condition, race/ethnicity, sex). Going forward, it will also be important for non-RCT evaluations to include a control group to elevate the rigor of the study design and to isolate true impact of the intervention. Such studies are happening with less frequency making rigorous comparisons nearly impossible. Improved knowledge in these areas and others may advance the strategic framework on multiple chronic conditions proposed by the US Department of Health and Human Services, which seeks to change how chronic illnesses are addressed in the United States (50).

Our review has limitations. Although we relied exclusively on peer reviewed publications, some studies had methodological flaws such as 1) suboptimal study designs (eg, no control group), which may lead to biased results or 2) small sample sizes, which limit the generalizability of study results (only 3 of the 27 studies had samples with more than 1,000 people). The number of unsuccessful programs may be even larger because of publication bias.

The outcomes being measured are not consistent across the various studies. Our study was restricted to articles published in English and only focused on programs operating in the United States. Our study was also restricted to interventions targeted to patients with multiple chronic conditions, and therefore excluded numerous successful studies that were limited to a single disease. The diversity of disease combinations included in these studies limits the generalizability of these results. Lastly, our focus on the peer-reviewed literature may omit relevant evaluations that were published elsewhere, for example, in reports.

This study has numerous strengths. We included a range of intervention models with different patient populations, across all ages, all targeting people with multiple chronic conditions. Some prior reviews focused on 1 model only (eg, comprehensive geriatric assessment) or 1 population only (eg, Medicare recipients). Robust review methods were used, including paired reviewers. The findings from this review can guide research and implementation strategies about the most appropriate models of care for people with multiple chronic conditions.

As large-scale changes to the health care delivery system continue, more rigorous evaluations of programs are needed so that program developers can be better informed about what is most likely to be effective in reducing spending and improving outcomes for this important and growing population.

## Appendix

This appendix is available for download as a Microsoft Word document.
