# Erratum, Vol. 12, November 12 Release

**DOI:** 10.5888/pcd13.150275e

**Published:** 2016-03-03

In the article “Systematic Review of Programs Treating High-Need and High-Cost People With Multiple Chronic Diseases or Disabilities in the United States, 2008–2014,” the author inadvertently listed a coauthor’s name incorrectly. Eva Chang, PhD, should have appeared as Eva DuGoff, PhD, MPP. The author list was corrected on February 12, 2016, and appears at http://www.cdc.gov/pcd/issues/2015/15_0275.htm. We regret any confusion this error may have caused. 

