# Supplementary material for: Systematic Review of Programs Treating High-Need and High-Cost People With Multiple Chronic Diseases or Disabilities in the United States, 2008–2014
Source: Prev Chronic Dis. 2015 Nov 12;12:E197. doi: 10.5888/pcd12.150275 (PMC4651160; doi:10.5888/pcd12.150275)
Supplement: Supplementary file 1 [file 15_0275_Appendix.docx]

# APPENDIX

## Appendix Table A1: Highly Successful Patient Satisfaction Outcomes for Programs Treating High Cost High Needs People (N=3)

| **Study, Year** | **Patient Satisfaction Outcomes** | **Reported Outcome at End of Follow-Up** | | **Statistical Measure of Effect** | **Test of Significance** |
| --- | --- | --- | --- | --- | --- |
|  |  | **Control** | **Intervention Group** |  |  |
| Friedman, 2009^a^ | Patient improved health (mean scale) | 3.45 | 3.61 | t=2.24 | p< .05 |
|  | Patient satisfaction with nurse tool (mean scale) | 3.38 | 3.6 | t=2.26 | p< .05 |
|  | Patient satisfaction with nurse relationship (mean scale) | 4.21 | 4.35 | t=2.73 | p< .05 |
|  | Patient satisfaction with primary care provider/health provider (mean scale) | 3.35 | 3.56 | t=2.32 | p< .05 |
|  | Patient general satisfaction with nurse intervention (mean scale) | 3.72 | 4.0 | t=3.9 | p< .05 |
|  | Patient satisfaction with primary care partnership meeting (mean scale) | 3.35 | 3.73 | t=3.24 | p<.01 |
|  | Caregiver satisfaction with primary care provider/health provider (mean scale) | 3.64 | 3.77 | t=1.87 | p<.1 |
|  | Caregiver satisfaction with nurse help to reduce caregiver stress (mean scale) | 2.64 | 2.85 | t=2.17 | p< .05 |
| Gellis, 2012^b^ | SF-36 subscale general health(mean) | 41.5 | 48.4 | F=3.91 | p< .016 |
|  | SF-36 subscale social functioning(mean) | 46.4 | 56.3 | F=3.64 | p< .014 |
| Luptak, 2010 | Overall positive response to device (%) |  | 79.3 |  | NR |
|  | Did not have difficulty with device (%) |  | 94.6 |  | NR |
|  | Likely to very likely to continue to use device (%) |  | 78.5 |  | NR |
|  | Satisfied to very satisfied with device (%) |  | 86 |  | NR |
|  | Easy to very easy to use device (%) |  | 96.8 |  | NR |
|  | Improved communication between themselves and primary health care provider (%) |  | 49.5 |  | NR |
| ^a^No control group in this study. Values under “Control” represent 10 month follow-up and those under “Intervention” represent 20 month follow-up  ^b^ A difference between control and intervention groups was not found. Values under “Control” represent baseline data for the intervention and values under “Intervention: represent 3 month follow-up outcomes.  NR= not reported; NA=not applicable; SF-36= Medical Outcomes Study of Health Related Quality of Life; AOR= Adjusted odds ratio; CE=Coefficient estimate  ^c^ Control represent baseline data for the intervention group and the intervention group represents outcomes at 12 months. | | | | | |

## Appendix Table A2: Highly Successful Clinical Outcomes for Programs Treating High Cost High Needs People (N=14)

| **Study, Year** | **Clinical Outcomes** | **Reported Outcome at End of Follow-Up** | | **Intervention Group** | **Statistical Measure of Effect** | **Test of Significance** |
| --- | --- | --- | --- | --- | --- | --- |
|  |  | **Control** | **Intervention Group** |  |  |  |
| Alexopoulous, 2011 | 12-item World Health Organization Disability Assessment Schedule II (WHODAS II) weeks 12 to 36 (disability during treatment) | NA | -2.14 |  | t=-2.46 | p=.01 |
| Blank, 2011 | Difference on the log of viral load at 12 months compared to baseline | NA | -.361 |  | t=1.30 | p<.001 |
| Comart, 2013 | Depression (effect estimate difference per quarter of control vs. intervention group) | NA | -.11 |  |  | p=.031 |
|  | Delirium | NA | -.05 |  |  | p=.440 |
|  | Falls | NA | -.06 |  |  | p=.374 |
|  | Pains | NA | -.06 |  |  | p=.341 |
|  | Skin ulcers | NA | -.07 |  |  | p=.322 |
|  | Shortness of breath | NA | -.11 |  |  | p=.141 |
|  | Weight loss | NA | .03 |  |  | p=.720 |
|  | Number of medications | NA | -.09 |  |  | p=.213 |
|  | Physician orders | NA | -.16 |  |  | p=.130 |
|  | Hospitalizations | NA | -.08 |  |  | p=.363 |
|  | ER visits | NA | -.92 |  |  | p<.001 |
|  | Composite of all outcomes | NA | -.27 |  |  | p<.001 |
| Gellis, 2012 | Depression Post-treatment(mean PHQ-9 score) | 15.2 | 14.9 |  |  | p<.008 |
|  | Depression Baseline(mean PHQ-9 score) | 13.6 | 7.4 |  |  |  |
|  | Hemoglobin A1c change from baseline at 3 months(mean) | -0.07 | -0.4^b^ | -0.44^c^ | F=3.47 | p=.03^d^ |
|  | Hemoglobin A1c change from baseline at 12 months(mean) | -0.33 | -0.17^b^ | -0.19^c^ | F=.43 | p=.65^d^ |
|  | Systolic blood pressure change from baseline to 12 months(mean scale) | 3.34 | 0.76^b^ | -4.92^c^ | F=3.84 | p=.02^d^ |
| Gutgsell, 2013 | Pain(NRS mean score) Pre | 6.41 | 6.69 |  | NR | p<.0001 |
|  | Pain(NRS mean score) Post | 5.86 | 4.74 |  |  |  |
|  | Pain(FPS mean) | 2.3 | 2.38 |  | NR | p<.0001 |
|  | Pain(FPS mean) | 2.19 | 1.76 |  |  |  |
| Jerant, 2009 | Illness Management Self-efficacy Score at 6 weeks (mean) | 7.2 | 7.6 | 7.2 | NR | p=.001 |
|  | Illness Management Self-efficacy Score at 6 months (mean) | 7.2 | 7.5 | 7.3 | NR | p=.04 |
|  | Illness Management Self-efficacy Score at 1 year(mean) | 7.2 | 7.4 | 7.2 | NR | NR |
|  | Physical component summary score (PCS-36) at 6 weeks (mean) | 37.3 | 34.9 | 36.3 | NR | NR |
|  | PCS-36 at 6 months (mean) | 37.2 | 36.2 | 37.5 | NR | NR |
|  | PCS-36 at 1 year (mean) | 37.0 | 35.1 | 36.4 | NR | NR |
|  | Mental component summary score (MCS-36) at 6 weeks (mean) | 48.6 | 51.6 | 48.6 | NR | NR |
|  | MCS at 6 months (mean) | 48.0 | 49.6 | 48.0 | NR | NR |
|  | MCS at 1 year (mean) | 48.5 | 51.2 | 48.5 | NR | NR |
| Kuo, 2013 | Functional Status RII (Child Functioning Scale | 76.2^c^ | 76.6^c^ |  |  | p=.76 |
|  | Impact on family: Social | 23.2 ^c^ | 26.0^c^ |  |  | p=.06 |
|  | Impact on family: Personal | 14.6^c^ | 14.7^c^ |  |  | p=.84 |
|  | Parent Support Scale | 3.6^c^ | 4.4^c^ |  |  | p=.13 |
|  | SF-12 Health-Related Quality of Life, physical subscale | 49.1^c^ | 46.4^c^ |  |  | p<.01 |
|  | SF-12 Health-Related Quality of Life, mental subscale | 45.4^c^ | 45.5^c^ |  |  | p=.92 |
| Kiosses, 2011^a^ | Depression(change in HDRS-24 score at 12 weeks)- Mr. X | NA | -14 |  | NA | NA |
|  | Depression(change in HDRS-24 score at 12 weeks)- Mrs. Y | NA | -10 |  | NA | NA |
|  | Disability(change in SDS score at 12 weeks)- Mr. X | NA | -4 |  | NA | NA |
|  | Disability(change in SDS score at 12 weeks)- Mrs. Y | NA | -8 |  | NA | NA |
| Li, 2012 | ADL shopping preparation(mean subscale) | | | | AOR=.33 | p=.02 |
|  | ADL meal preparation(mean subscale) | | | | AOR=.55 | p=.08 |
| Moggi, 2010 | Abstinence from drugs and alcohol (% yes) | 42.4^d^ | 34.8 ^d^ |  | F=.84 | NR |
|  | Substance use problems (mean) | 8.1^d^ | 15.2^d^ |  | F=5.05 | p<.05 |
|  | Emotional distress (mean) | 7.9^d^ | 17.6^d^ |  | F=19.88 | p<.01 |
|  | Psychotic symptoms (mean) | 5.8^d^ | 11.5^d^ |  | F=11.29 | p<.01 |
| Ornstein,2013^b^ | Pain(% symptom free) | 25 | 27.08 |  | NR | p<.05 |
|  | Depression(% symptom free) | 57.58 | 50 |  | NR | p<.05 |
|  | Anxiety(% symptom free) | 58.6 | 59.3 |  |  | p<.05 |
|  | Tiredness(% symptom free) | 45.1 | 47.5 |  |  | p<.05 |
| Petry, 2012 | Duration of drug abstinence(weeks) | 2.6 | 5 |  | F=4.22 | p=.04 |
|  | Unexcused abstinences | 8.1 | 4.3 |  |  | p<.002 |
| Wakefield, 2011 | Hemoglobin A1c change from baseline at 6 months(mean) | -0.07 | -0.4^e^ | -0.44 | F=3.47 | p=.03 |
|  | Hemoglobin A1c change from baseline at 12 months(mean) | -0.33 | -0.17^e^ | -0.19 | F=.43 | p=.65 |
|  | Systolic blood pressure change from baseline to 12 months(mean scale) | 3.34 | 0.76^e^ | -4.92 | F=3.84 | p=.02 |
| Wakefield, 2012 | Self-efficacy scores from baseline at 6 months | 8.1 | 8.1 | 7.7 |  | p=.09 |
|  | Self-efficacy scores from baseline at 12 months | 8.3 | 8.3 | 7.8 |  | p=.19 |
|  | Medication-taking adherence from baseline at 6 months | 99.6 | 99.8 | 99.6 |  | p=.79 |
|  | Medication-taking adherence from baseline at 12 months | 98.9 | 99.7 | 100 ^f^ |  | p=.20 |
| ^a^Article reported intervention effects on 2 patients. Statistical tests not appropriate.  ^b^No control group in this study. Values under “Control” represent 10 month follow-up and those under “Intervention” represent 20 month follow-up  NR= not reported; NA=not applicable; PHQ-9= Patient Health Questionnaire- 9 items; ADL= Activities of Daily Living; IADL= Instrumental Activities of Daily Living; NRS= Numeric Ration System; FACLS= Face, Legs, Activity, Cry, Consolability Scale; HDRS= Hamilton Depression Rating Scale-24 items; SF-12= Short Form-12 items; SDS= Sheehan Disability Scale; AOR= Adjusted odds ratio; CE=Coefficient estimate  ^c^ Control represent baseline data for the intervention group and the intervention group represents outcomes at 12 months.  ^d^ Control represent patients in Switzerland and intervention group represents patients in the U.S. | | | | | | |

## Appendix Table A3: Highly Successful Health Care Utilization Outcomes for Programs Treating High Cost High Needs People (N=13)

| **Study, Year** | **Health Care Utilization Outcomes with High Success** | **Reported Outcome at End of Follow-Up** | | **Statistical Measure of Effect** | **Test of Significance** |
| --- | --- | --- | --- | --- | --- |
|  |  | **Control** | **Intervention Group** |  |  |
| Barett, 2010^a^ | ED visits | 185 | 34 | NR | <.001 |
|  | all-cause hospitalization | 208 | 53 | NR | <.001 |
| Boult, 2011 | Home health care episodes (mean annual per capita use) | 1.30 | .99 | AOR= .70 | CI= (.53-.93) |
|  | Hospital admissions | .72 | .70 | AOR= 1.01 | CI= (.83-1.23) |
|  | 30-Day readmissions | .17 | .13 | AOR=.79 | CI= (.53-1.16) |
|  | Hospital days | 4.49 | 4.26 | AOR=1.00 | CI= (.77-1.30) |
|  | Skilled nursing facility admissions | .25 | .20 | AOR=.92 | CI= (.60-1.40) |
|  | Skilled nursing facility days | 4.03 | 2.84 | AOR=.84 | CI= (.48-1.47) |
|  | Emergency department visits | .44 | .44 | AOR=1.04 | CI= (.81-1.34) |
|  | Primary Care visits | 9.88 | 9.89 | AOR=1.02 | CI= (.91-1.14) |
|  | Specialist visits | 8.49 | 9.04 | AOR=1.07 | CI= (.93-1.23) |
| Casey, 2011^a^ | Overall cost to Medicaid(mean, $) | 4703 | 3524 | NR | p=0.001 |
|  | LOS | 1.25 | 2.25 | NR | p=0.001 |
|  | LOS per hospitalization | 14.5 | 10 | NR | p=0.01 |
| Comart, 2013 | Hospitalizations | NA | -.08 |  | p=.363 |
|  | ER visits | NA | -.92 |  | p<.001 |
| De Jonge, 2014 | Hospitalizations (% reduction) | NA | 9 |  | p=.001 |
|  | ED visits (% reduction) | NA | 10 |  | p=.001 |
|  | Specialist visits (% reduction) | NA | 23 |  | p=.001 |
|  | Generalist visits (% gain) | NA | 105 |  | p<.001 |
|  | Total Medicare Costs ($) | 50,978 | 44,455 |  | p=0.1 |
| Edelman,2010 | ED visit(mean visits/ pt-yr) | 1.3 | 0.9 | NR | p<.001 |
|  | Primary care visits(mean vistis/ pt-yr) | 6.2 | 5.3 | NR | p=.01 |
| Edes, 2014 | Medicare hospital days | 4,511 | 4,161 |  | p<.001 |
|  | Medicare skilled nursing facility days | 5,559 | 5,594 |  | p=.68 |
|  | Total Medicare cost per patient, $ (6 months) | 4,025 | 3,590 |  | p<.001 |
|  | VA hospital days | 8,877 | 4,339 |  | p<.001 |
|  | Total VA costs per patient, $ (6 months) | 19,234 | 13,822 |  | p<.001 |
|  | VA and Medicare hospital admissions per 100 patients-months | 15.7 | 11.7 |  | p<.001 |
|  | VA and Medicare hospital days | 13,388 | 8,500 |  | p<.001 |
| Kuo, 2011 | Need Help with care coordination (%) | 78.3 ^c^ | 31.3 ^c^ |  | p<.001 |
|  | Child has a written care plan (%) | 52.9 ^c^ | 84.9 ^c^ |  | p<.001 |
|  | Receive info about family support (%) | 45.7 ^c^ | 55.3 ^c^ |  | p=.14 |
|  | Receive info about current research (%) | 37.5 ^c^ | 33.7 ^c^ |  | p=.52 |
|  | Care team helps with understanding child’s emotional needs (%) | 54.8 ^c^ | 49.4 ^c^ |  | p=.46 |
|  | Care team shows concern about impact of health on family (%) | 62.7 ^c^ | 62.7 ^c^ |  | p=1 |
|  | No shows | NA | NR | NR | p<.01 |
| Moggi, 2010 | Remission (% yes) | 14.1 ^d^ | 22.7 ^d^ | F=1.49 | NR |
|  | Hospitalization (% yes) | 20.6 ^d^ | 39.4 ^d^ | F=6.83 | p<.01 |
| North, 2008 | Direct Cost Savings ($) | NA | NR |  | p<.01^b^ |
|  | ED visits | NA | NR | NR | p<.01 |
| O'Toole, 2009 | All-cause hospitalization (mean admission/ person) | 0.47 | 0.15 |  | p=.02 |
|  | Non-emergent ED visits | 38.6 | 18.5 |  | p<.01 |
|  | ER visits(summary score) | | | CE=0.521 | p<.01 |
| Ouslander, 2009 | Preventable hospitalizations (% reduction) | 77 | 49 | NR | NR |
| Takahashi, 2013 | Inpatient hospitalization (%) | 10.5 | 0.0 |  | p=0.17 |
|  | ER visits (%) | 31.6 | 11.8 |  | p=0.37 |
| ^a^ High success is defined as greater than 1 statistically significant patient satisfaction outcome.  ^b^ P-value compares mean absolute reduction across all groups. ^c^ low continuity intervention ^d^ moderate continuity intervention ^e^ high continuity intervention ^f^ compares low continuity to high ^g^ compares moderate continuity to high ^h^ No control group in this study. Values under “Control” represent baseline assessment and those under “Intervention” represent post-intervention follow-up ^I^ P-values compare overall pre post intervention assessment  ^I^ compares intervention to long term care facility  CI= confidence interval; Pt= patient; Yr= year; ED= emergency Department; LOS= Length of Stay; AOR= Adjusted odds ratio;  CE=Coefficient estimate  ^c^ Control represent baseline data for the intervention group and the intervention group represents outcomes at 12 months.  ^d^ Control represent patients in Switzerland and intervention group represents patients in the U.S. | | | | | |
